# Supplementary material for: A Neural Network Approach to Identify Glioblastoma Progression Phenotype from Multimodal MRI
Source: Cancers (Basel). 2021 Apr 21;13(9):2006. doi: 10.3390/cancers13092006 (PMC8121245; doi:10.3390/cancers13092006)
Supplement: Supplementary file 1 [file cancers-13-02006-s001.zip › cancers-1176346-supplementary.pdf]

Supplementary Material: A Neural Network Approach to Identify Glioblastoma Progression Phenotype from Multimodal MRI

Jiun-Lin Yan, Cheng-Hong Toh, Li Ko, Kuo-Chen Wei and Pin-Yuan Chen

Text S1: MR Imaging Processing

Reposition and reorientation of the images were done prior to the imaging processing.

1. Brain Extraction

Brain extraction by using FMRIB Software Library (FSL) function ("bet") was automatically followed by manual correction [1] with the following script: bet <input> <output> –f 0.05 –m

2. DTI Imaging Procession and DTI analysis

DTI images were processed using FSL version 5.0.0 (www.fmrib.ox.ac.uk/fsl) [2]. Eddy current can occur nearby the conductor whenever magnetic field changes. This effect can cause unwilling stretch or shearing of the images due to the changing of the magnetic field. The effect of eddy current depends on the rate of changes of the magnetic field, therefore in DTI which uses the fast echo-planner imaging can commonly have this artifact. Therefore, eddy current correction is needed before processing by using "eddy_correct" function in FSL FMRIB's diffusion toolbox (FDT).

- Eddy current correction: eddy_correct DTI.nii data 0
- Manual correction brain extraction with fslview: output: DTI_brain_mask_m
- Create binary mask for further calculation
- Fslmaths DTI_brain_mask_m.nii.gz –div DTI_brain_mask_m.nii.gz DTI_brain_mask_m_bin.nii.gz
- Create extracted brain with manual corrected mask: fslmaths DTI.nii –mul DTI_brain _mask_m_bin.nii.gz DTI_brain_m.nii.gz
- DTI analysis: dtifit -k data -m DTI_brain_m -r DTI.bvecs -b DTI.bvals -o dti

Further DTI parameters were then calculated by using FSL "dtifit" function. Outputs of the DTI processing include:

FA Fractional anisotropy
L1, L2, L3 the tensor eigenvalue (magnitude)
MD Mean diffusivity
MO Mode of the anisotropy
SO raw T2 signal with no diffusion weighting (b0)
V1, V2, V3 the tensor eigenvector

ADC was generated directly from scanner with an inline calculation, utilizing b-values 0–1000. Further calculation of the p and q were proceeded by using "fslmaths" function under FSL terminal with the below equation as described previously [3].

$$p = MD \times 1.732 \quad (1)$$

$$q = \sqrt{(\lambda 1 - D) \times 2 + (\lambda 2 - D) \times 2 + (\lambda 3 - D) \times 2} \quad (2)$$

Create different maps

```
fslmaths dti_MD -mul 1.732 DTI_p
fslmaths dti_L1 -sub dti_MD -sqr DTI_L1diff
fslmaths dti_L2 -sub dti_MD -sqr DTI_L2diff
fslmaths dti_L3 -sub dti_MD -sqr DTI_L3diff
fslmaths dti_L1diff -add dti_L2diff -add dti_L3diff -sqrt DTI_q
```

3. Imaging Coregistration

All MRI data were coregistered to pre-operative T1 with contrast MRI. Images coregistrations were done by using "FLIRT" function in FSL with the following script. within the same scan time point between different sequences. flirt -ref <reference image> -in <input images> -out <output of FLIRT> -omat <reference transformation> -cost normmi -searchrx -90 90 -searchry -90 90 -searchrz -90 90 -dof 12 -interp trilinear

4. Definition of the ROIs and the progression patterns

ROIs was defined by the contrast enhanced lesion of the pre-operative MRI. This manual selected ROIs were done by using 3D slicer (http://www.slicer.org) [4]

Table S1. Radiomics features.

1st order (18)	Shape (13)	GLCM (23)	GLDM (14)	GLRLM (16)	GLSZM (16)	NGTDM (5)
Interquartile Range	Maximum 3D Diameter	Joint Average	Gray Level Variance	Short Run Low Gray Level Emphasis	Gray Level Variance	Coarseness
Skewness	Maximum 2D Diameter Slice	Sum Average	High Gray Level Emphasis	Gray Level Variance	Zone Variance	Complexity
Uniformity	Sphericity	Joint Entropy	Dependence Entropy	Low Gray Level Run Emphasis	Gray Level Non-Uniformity Normalized	Strength
Median	Minor Axis	Cluster Shade	Dependence Non-Uniformity	Gray Level Non-Uniformity Normalized	Size Zone Non-Uniformity Normalized	Contrast
Energy	Elongation	Maximum Probability	Gray Level Non-Uniformity	Run Variance	Size Zone Non-Uniformity	Busyness
Robust Mean Absolute Deviation	Surface Volume Ratio	Idmn	Small Dependence Emphasis	Gray Level Non-Uniformity	Gray Level Non-Uniformity	
Mean Absolute Deviation	Volume	Joint Energy	Small Dependence High Gray Level Emphasis	Long Run Emphasis	Large Area Emphasis	
Total Energy	Major Axis	Contrast	Dependence Non-Uniformity Normalized	Short Run High Gray Level Emphasis	Small Area High Gray Level Emphasis	
Maximum	Surface Area	Difference Entropy	Large Dependence Emphasis	Run Length Non-Uniformity	Zone Percentage	
Root Mean Squared	Flatness	Inverse Variance	Large Dependence Low Gray Level Emphasis	Short Run Emphasis	Large Area Low Gray Level Emphasis	
90 Percentile	Least Axis	Difference Variance	Dependence Variance	Long Run High Gray Level Emphasis	Large Area High Gray Level Emphasis	
Minimum	Maximum 2D Diameter Column	Idn	Large Dependence High Gray Level Emphasis	Run Percentage	High Gray Level Zone Emphasis	
Entropy	Maximum 2D Diameter Row	Idm	Small Dependence Low Gray Level Emphasis	Long Run Low Gray Level Emphasis	Small Area Emphasis	
Range		Correlation	Low Gray Level Emphasis	Run Entropy	Low Gray Level Zone Emphasis	
Variance		Autocorrelation		High Gray Level Run Emphasis	Zone Entropy	
Kurtosis		Sum Entropy		Run Length Non-Uniformity Normalized	Small Area Low Gray Level Emphasis	
Mean		Sum Squares				
		Cluster Prominence				
		Imc2				
		Imc1				
		Difference Average				
		Id				
		Cluster Tendency				

References:

1. Smith, S.M. Fast robust automated brain extraction. *Hum. Brain Mapp.* **2002**, *17*, 143–155, doi:10.1002/hbm.10062.
2. Jenkinson, M.; Beckmann, C.F.; Behrens, T.E.; Woolrich, M.W.; Smith, S.M. FSL. *NeuroImage* **2012**, *62*, 782–790, doi:10.1016/j.neuroimage.2011.09.015.
3. Peña, A.; Green, H.A.L.; A Carpenter, T.; Price, S.J.; Pickard, J.D.; Gillard, J.H. Enhanced visualization and quantification of magnetic resonance diffusion tensor imaging using thep:qtensor decomposition. *Br. J. Radiol.* **2006**, *79*, 101–109, doi:10.1259/bjr/24908512.
4. Fedorov, A.; Beichel, R.; Kalpathy-Cramer, J.; Finet, J.; Fillion-Robin, J.-C.; Pujol, S.; Bauer, C.; Jennings, M.; Fennessy, F.; Sonka, M.; et al. 3D Slicer as an image computing platform for the Quantitative Imaging Network. *Magn. Reson. Imaging* **2012**, *30*, 1323–1341, doi:10.1016/j.mri.2012.05.001.
